# Supplementary material for: Preferences for oral and injectable PrEP among qualitative sub-study participants in HPTN 084
Source: PLoS One. 2024 Oct 23;19(10):e0309811. doi: 10.1371/journal.pone.0309811 (PMC11498703; doi:10.1371/journal.pone.0309811)
Supplement: S1 Table — (DOCX) [file pone.0309811.s001.docx]

# **Supporting Information**

**S1 Table. Injectable PrEP likes and dislikes at baseline**

|  | **All**  **(n=76)** | **Malawi**  **(n=20)** | **South Africa**  **(n=20)** | **Uganda**  **(n=17)** | **Zimbabwe**  **(n=19)** |
| --- | --- | --- | --- | --- | --- |
| **What do you think you might like?**  Nothing  May protect against HIV  Easier to use than other methods  May provide longer term protection  Can be used discreetly, without a partner’s knowledge  Is administered by a healthcare provider  Does not interrupt sex  Other (if 5% or more)  Prefer not to answer | %  3  55  58  51  42  34  30  3  5 | %  0  35  60  40  55  35  20  5  10 | %  5  55  50  35  20  20  20  0  0 | %  0  71  59  76  41  35  41  6  12 | %  5  63  63  58  53  47  42  0  0 |
| **What concerns did you have?**  None  May not protect against HIV  May be painful  May cause side effects  Not reversible  Cannot be used discreetly, without a partner’s knowledge  Cost may be unaffordable  Other  Prefer not to answer | %  33  17  32  28  24  5  20  3  7 | %  25  25  40  15  20  0  15  0  10 | %  20  20  30  35  20  10  15  5  10 | %  29  12  35  35  29  0  35  6  6 | %  58  11  21  26  26  11  16  0  0 |
