# Supplementary material for: Preferences for oral and injectable PrEP among qualitative sub-study participants in HPTN 084
Source: PLoS One. 2024 Oct 23;19(10):e0309811. doi: 10.1371/journal.pone.0309811 (PMC11498703; doi:10.1371/journal.pone.0309811)
Supplement: S2 Table — (DOCX) [file pone.0309811.s002.docx]

**S2 Table. Oral PrEP likes and dislikes at baseline**

|  | **All**  **(n=76)** | **Malawi**  **(n=20)** | **South Africa**  **(n=20)** | **Uganda**  **(n=17)** | **Zimbabwe**  **(n=19)** |
| --- | --- | --- | --- | --- | --- |
| **What do you think you might like?**  Nothing  May protect against HIV  Easier to use than other methods  Can be used discreetly, without a partner’s knowledge  Does not interrupt sex  Easily reversible  Other  Prefer not to answer | %  14  63  38  33  33  21  1  4 | %  25  35  25  30  25  20  0  10 | %  25  70  15  20  15  10  0  0 | %  0  82  59  53  47  29  6  6 | %  5  68  58  32  47  26  0  0 |
| **What concerns did you have?**  None  May not protect against HIV  May cause harmful side effects  Requires taking a daily pill  Cannot be used discreetly, without a partner’s knowledge  Cost may be unaffordable  Other  Prefer not to answer | %  22  22  33  45  17  11  3  3 | %  5  30  20  45  25  5  5  5 | %  10  25  50  60  15  5  0  0 | %  29  24  29  41  6  18  6  6 | %  47  11  32  32  21  16  0  0 |
