# Supplementary material for: Preferences for oral and injectable PrEP among qualitative sub-study participants in HPTN 084
Source: PLoS One. 2024 Oct 23;19(10):e0309811. doi: 10.1371/journal.pone.0309811 (PMC11498703; doi:10.1371/journal.pone.0309811)
Supplement: Attachment — Submitted filename: Response to Reviewers_05242024.docx [file pone.0309811.s004.docx]

Responses to Reviewer:

**Reviewer #1:** The acceptability and individual preference of PrEP modalities will become more and more important as more PrEP products become available and research shifts to implementation. This work is a very important contribution highlighting the importance of considering not only stigma, but also practicability, short and long term side effects as well as monetary considerations.

My only major comment is that I would like to see a bit more quantitative analysis. For each pattern that is highlighted in the "findings" section, you should indicate how many respondents were asked a similar question. Sometimes this information is given and sometimes it is not or it is given only vaguely (about one third). I feel that we get a good quantitative breakdown of concerns prior to receiving injections, but it would be good to see how that compares with what was found.
I think this is especially important as these IDI transcripts will not be made publicly available due to justifiable privacy concerns.

**Response:** In response to this and reviewer 3’s comments, we have inserted additional numbers into several sections of findings. Qualitative researchers have different opinions about how to represent themes numerically, since not all participants are asked the same questions, or spontaneously raise topics in a standard way. We have tried to substantiate important themes with some numbers in ( ) without detracting too much from the qualitative descriptions.

Very minor comments:

Background
Line 64 “The jury is still out” suggest rephrasing to more precise language. Perhaps “numerous demonstration projects and implementation studies show that PrEP adherence among women is typically not sufficient to achieve complete protection” and citing Marrazzo (2024) on this point.
Generally, seems like some of these authors have done more work on this research question that should be highlighted in the introduction. Velloza et al (10.1002/jia2.25463) specifically addressed reasons for non-adherence on HPTN082.

**Response:** Thank you for this suggestion. We have modified the introduction to discuss varying adherence trajectories described by Marrazzo (2024) and the Celum and Velloza articles. See highlighted section below.

While some research suggests that women using products with known efficacy can and will be more adherent (16), a pooled analysis of 11 post-approval PrEP studies conducted in cisgender women identified varying patterns of adherence over time (17). Based on a trajectory modelling approach that utilized available adherence data (including objective measures such as dried blood spots or tenofovir concentration measures, or more subjective measures like pill counts or self-reports) from almost 3000 women, about 40% of women reported consistent use (19%) or consistently high use (22% reported 4-6 pills per week) of PrEP up to 96 weeks of use. An equal proportion of women (39%) across these studies showed decreasing adherence over time, while about 21% reported consistently low adherence (17). Several smaller demonstration and implementation studies shed further light on these adherence patterns. For example, in a recent retrospective cohort study of 262 female PrEP initiators in Kisumu, Kenya, continuation dropped off significantly, with only 12% documented as having refilled their PrEP pills after 6 months (18, 19). Similarly, in a PrEP implementation study (HPTN 082) among 451 young women from South Africa and Zimbabwe, the majority (55%) had lapses in PrEP refills over a period of i12 months and only 21-22% were identified as highly adherent at six months (20). Women randomized to the advanced adherence arm of the study who reported at least one risk factor during a study visit were also more likely to have detectable drug on board, leading researchers to theorize that some young women might try to align their PrEP use with seasons of perceived risk (21). These social barriers continue to exist for women and make daily product use challenging.

Findings
Line 196 “By far, women appreciated the ease and convenience of a long-acting formulation” relative to an oral formulation?

**Response:** Yes, thank you. We have clarified.

**Reviewer #2**: This is a well written and interesting mixed methods manuscript that is important contribution to the literature as long-acting antiretrovirals are implemented in southern Africa. I only have minor comments for the authors to consider.
Introduction
Would recommend that the authors not use the trade names for TDF/FTC and TAF/FTC through out the manuscript. TDF is defined on line 45, but TAF/FTC is not (line 48).

**Response:** We have added the acronym for TAF/FTC in line 48. I have also removed the word “truvada” except for initial introduction.

Line 64: Would consider revising “the jury is still out” to something more descriptive such as “it has not been full evaluated”.

**Response:** Thank you for this suggestion. We have expanded this section per reviewer 1 suggestions and removed this phrase. (See above response.)

Line 70: Would include the full enrolled cohort, and not “> 3,200”

**Response:** Thank you. We have edited to read “3224”.

Methods
The methods are clear and outline the qualitative and quantitative approach for this analysis.

**Response:** Thank you.

Results
The results are well written and clear. The section on injection site pain was a little confusion, which seems to be secondary to the wording of the second sentence on lines 225-226 Perhaps change to “Some participants described the pain as easily tolerable”.

**Response:** Thank you for pointing out the need for some clarification. We have revised as follows:

For example, only a few participants randomized to the CAB arm, but many more in the TDF/FTC arm (5 versus 20), suggested that injectable pain was easily tolerated….

In contrast, many women described moderate to significant levels of pain, with some differences by arm.

Discussion
The discussion is well written and clear.

**Reviewer #3:** This manuscript used survey and qualitative data from a four-country sub-study in the HPTN 084 study to examine oral and injectable PrEP acceptability and considerations for CAB access among African women.

One of the key findings is “Participants overwhelmingly preferred injections to pills, ap- preciating the ease, convenience, and privacy of a long-acting formulation. ” However, such a finding is not fully demonstrated in the FINDINGS section. Specifically, the only relevant description is simply one sentence “Regardless of study arm, most women in the sub-study pre- ferred injections to daily pills when asked to indicate their preference; they often explained that the injection would be harder to forget to take and meant fewer clinic visits. ” I would suggest to include more details (e.g., proportion of women that prefer injection, quotes on forgetting taking PrEP and clinic visits) to strengthen the statement.

**Response:** We have added proportion of women who clearly preferred CAB to oral PrEP. (A number of women indicated that either product would be good.) We didn’t include additional quotes because similar quotes can be found in other sections.

Some other comments:
1. Abstract: what do you mean by “A study statistician identified participants’ assigned study arm”?

**Response:** The HPTN uses SCHARP – a statistical center in Seattle to conduct analyses. In order to stay blinded to study arm while conducting analysis, we requested that a study statistician from SCHARP draw up the lists of PTIDs, ensuring some balance across sites and arms, for the local study teams to then invite to participate.

2. Are the CPs also representative of early, mid-trial, or later entry? It is not clear from the description in the manuscript.

**Response:** the CP IDs were drawn from across these different time points. However, we did not formally try to analyze data by study period, since the comparisons would consist of very small numbers.

3. The SCs received up to two serial interviews. When are those interviews taken? Line 126: “Qualitative analyses presented in this paper focus on the first and second IDIs, both of which were conducted prior to participants’ ability to switch or remain on their current active product, i.e., during the blinded and unblinded periods of the study.” Is this statement also correct for the SCs?

**Response:** Thank you for this clarifying question. The statement is only applicable to the CPs, who took part in up to 3 different interviews. The final interview took place after participants were able to choose – and thus able to change the product they were using. Findings from those 3^rd^ interviews will be presented in a subsequent paper. I have clarified that the first two IDIs of both CPs and SCs were included in this analysis.

4. Line 291: “Less frequently (but more often than for injections), women listed an array 1
of side effects they attributed to oral pill use. ” What is the frequency for side effect for those randomized to the CAB arm? Can you add the number to Line 271?

**Response:** Thank you for this comment. We have indicated the proportion of side effects for injectables in the section above (10 of 76) and amended the oral product experiences as below:

Additionally, more than half of participants (41 of 76) described encountering some problems with study pill adherence.

5. Minor comments:
• Line 99: “from” should be removed?

**Response:** No, I believe “from” should stay. We created a list and then selected PTIDs from the list.

• Line 239: “single” is used for this women instead of “not married” by others.

**Response:** Thanks for pointing out this inconsistency. Now revised.
